# Cellular functions and biomedical applications of circular RNAs

**DOI:** 10.3724/abbs.2024241

**Published:** 2024-12-24

**Authors:** Zheyu Zhang, Zefeng Wang

**Affiliations:** 1 CAS Key Laboratory of Computational Biology Chinese Academy of Sciences Shanghai 200031 China; 2 Shool of Life Science Southern University of Science and Technology Shenzhen 518055 China

**Keywords:** circRNA, circRNA translation, circRNA synthesis, circRNA delivery, circRNA biomedical applications.

## Abstract

Circular RNAs (circRNAs) have emerged as a large class of stable and conserved RNAs that are derived primarily from back-splicing of pre-mRNAs and expressed in a cell- and tissue-specific fashion. Recent studies have indicated that a subset of circRNAs may undergo translation through cap-independent pathways mediated by internal ribosome entry sites (IRESs), m6A modifications, or IRES-like short elements. Considering the stability and low immunogenicity of circRNAs, *in vitro* transcribed circRNAs hold great promise in biomedical applications. In this review, we briefly discuss the noncoding and coding functions of circRNAs in cells, as well as the methods for the *in vitro* synthesis of circRNAs and current advances in the applications of circRNAs in biomedicine.

## Introduction

Circular RNAs (circRNAs), unlike linear RNAs, are generated through the back-splicing of a linear RNA molecule, resulting in the formation of a covalently closed single-stranded RNA structure. This circular form of RNA was first identified in plant viroids in 1976 [1]. Eukaryotic circRNAs were subsequently discovered via electron microscopy in 1979 [2]. Owing to technological limitations in the last century, the biogenesis and molecular mechanisms of circRNAs have remained unclear for an extended period. As a result, most circRNAs have long been considered useless RNAs arising from incorrect transcription and alternative splicing.

Over the past decade, the development of new molecular biology techniques, high-throughput sequencing methods, and bioinformatics algorithms has significantly advanced our understanding of circRNAs, as well as other noncoding RNAs, which illustrates the crucial roles of circRNAs in complex physiological and pathological conditions, such as cardiovascular diseases [3], aging and age-related diseases [4], oncogenesis [5], and beyond.

CircRNAs are generally generated from back-splicing of pre-mRNAs in the nucleus or from intron self-splicing of small nuclear RNAs (snRNAs), mitochondrial RNAs, ribosomal RNAs (rRNAs), and transfer RNAs (tRNAs) [ 6, 7]. The back-splicing of pre-mRNAs mostly occurs posttranscriptionally and has low efficiency in cells [8]. A specific pathway for the nuclear export of circular RNA was also identified recently, which indicated that adaptors such as IGF2BP1 could directly bind with circRNAs to recruit Ran-GTP and exportin-2 to export circRNAs from the nucleus to the cytoplasm, demonstrating that circRNAs are exported via a mechanism that is analogous to protein export rather than mRNA export [9]. Compared with linear RNAs, circular RNAs are more resistant to cleavage by RNA exonucleases due to a lack of free ends and thus are generally more stable in cells [ 8, 10], which is desirable for the development of circRNA-based drugs.

Current studies have demonstrated that circRNAs may have a wide range of functions as noncoding RNAs, including functions as modulators of transcription and molecular sponges for microRNAs or RBPs. Certain circRNAs tend to form 16–26 bp duplexes after degradation and act as endogenous PKR inhibitors [11]. In addition, circRNAs can also act as mRNAs to direct protein synthesis through cap-independent translation, which can be driven by IRES elements, m6A modifications or IRES-like sequences [ 12– 14]. An increasing number of natural translatable circRNAs and their translation products have also been identified, providing a novel perspective on the diverse functions and potential biomedical applications of circRNAs [15] .

With the emergence of the clinical application of two mRNA vaccines against COVID-19 in 2021 (Pfizer-BioNTech’s BNT162b2 and Moderna’s mRNA-1273), RNA-based drugs and therapies have increasingly attracted the attention of the biomedical community [ 16, 17]. Recent studies have indicated that circRNAs exhibit greater stability and durability than modified linear mRNAs do, since they do not have exposed 5′ and 3′ ends. As a result, the proteins translated from these circRNAs also persist for a longer duration *in vivo* [18]. Furthermore, *in vitro*-transcribed purified circRNAs have lower immunogenicity without the need for base modifications, which are necessary for linear mRNA vaccines [19]. These findings highlight the potential for extensive applications of circRNAs in various biomedical fields.

In this review, we focus on the discussion of the cellular functions, synthesis methods, delivery strategies and potential applications of circRNAs. We discuss the roles and application of circRNA translation, briefly summarizing the cellular functions of circRNAs as noncoding RNAs.

## Cellular Functions of circRNAs

### Noncoding roles of circRNAs

Studies on circRNAs have significantly increased since 2013, when certain circRNAs were discovered to function as miRNA sponges to sequester miRNAs [20]. After sequencing and computational analyses of transcriptomes of human, mouse and nematode RNA, a subset of circRNAs were found to have tissue- or developmental-stage-specific expression patterns [21]. A human circRNA transcribed in the antisense direction to the cerebellar degeneration-related protein 1 transcript (CDR1as) was first found to harbor 63 conserved binding sites for miR-7 and was densely bound by miRNA effector complexes [20]. Further studies demonstrated that the circRNA CDR1as acts as a miR-7 sponge to impact midbrain development by increasing the mRNA levels of miR-7 targets [20]. Since then, several studies have confirmed that circRNAs may function as miRNA sponges, containing multiple miRNA binding sites and acting as competing endogenous RNAs (ceRNAs) to modulate miRNA accessibility to target mRNAs [7]. Despite the large body of research on circRNA/miRNA/mRNA competitive endogenous RNA (ceRNA) regulatory networks reported, questions persist about the mechanism involved because of the low abundance of most circRNAs and the restricted number of miRNA binding sites on them.

In addition to acting as miRNA sponges, recent studies have shown that circRNAs can play a variety of roles in the regulation of gene expression by interacting with other biomacromolecules, including DNAs, RNAs and proteins. The biological roles of such interactions range from modulating transcription in the nucleus to affecting translation in the cytoplasm. Here, we briefly summarize the noncoding roles of circRNAs in regulating gene expression with several examples. More detailed research findings can be found in other reviews [ 22, 23].

As an example of circRNAs interacting with DNAs, the circRNAs derived from exon 6 of the SEPALLATA3 ( *SEP3*) gene strongly bind to its cognate DNA locus, forming an RNA:DNA hybrid structure known as an R-loop. In contrast, the linear RNA equivalent bound significantly weakly to DNA. This R-loop structure leads to transcriptional pausing and an increase in the abundance of the cognate exon-skipped AS variant, thereby driving floral homeotic phenotypes [24].

In addition to interactions with DNAs and RNAs through complementary base pairing, certain circRNAs have been found to interact with proteins in various ways. In general, the binding of circRNAs to a specific protein usually inhibits the original function of its protein partner. Moreover, some circRNAs with multiple binding sites can bind to two different proteins, thereby cementing or dissociating interactions between the two binding proteins [22]. For example, circ-Foxo3 plays a crucial role in repressing apoptosis by suppressing p53 in breast carcinoma biopsies and in cancer cell lines. It can bind with both p53 and MDM2. Given that MDM2 is capable of polyubiquitinating p53, the circ-Foxo3-p53-MDM2 complex enhances the polyubiquitination of p53 by MDM2 and ultimately suppresses p53 in a proteasome-dependent manner [25] .

### Translation of circRNAs

**Figure 1:**
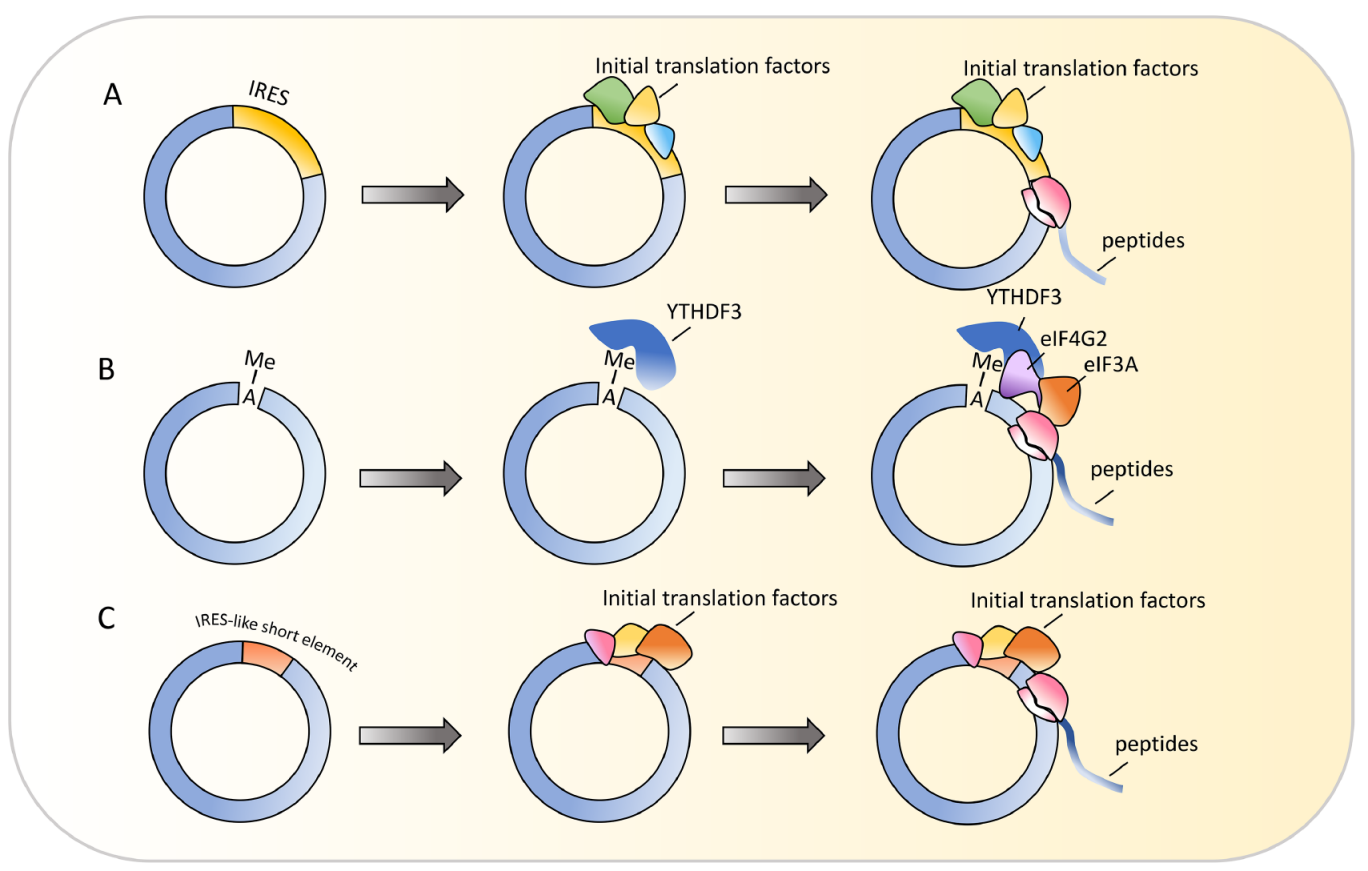
Figure 1 Cap-independent translation of circRNAs (A) Internal-ribosome entry site (IRES)-mediated cap-independent mechanism of circRNA translation. (B) m6A-mediated cap-independent mechanism of circRNA translation. (C) IRES-like element-mediated cap-independent mechanism of circRNA translation

Most eukaryotic mRNAs are translated through a cap-dependent mechanism under normal conditions. However, in response to stress conditions, an alternative translation mechanism known as cap-independent translation has long been known to initiate mRNA translation through the internal ribosome entry site (IRES) [26]. As covalently closed circles lacking m7G caps and poly-A tails, circRNAs cannot undergo cap-dependent translation to produce proteins even when they contain an open reading frame (ORF) with a start codon. Instead, recent studies have indicated that circRNAs can be translated in a cap-independent manner driven by internal ribosomal entry sites (IRESs) ( Figure 1A).

In 1995, it was first demonstrated that an *in vitro*-produced circular RNA containing an IRES from encephalomyocarditis virus (EMCV) could be translated into peptides when it was inoculated with rabbit reticulocyte lysate [27], suggesting that the translation of circRNAs is chemically possible. In 1998, an *in vitro* synthesized circRNA containing a GFP ORF was reported to direct GFP expression in *Escherichia coli* [28], and circRNAs containing an infinite ORF (ORF with no stop codons) produced extremely long protein chains in bacterial cells [28]. A subsequent study revealed a rolling cycle translation mechanism in which small circRNAs with infinite ORFs were translated more efficiently than their linear counterparts via an *E*. *coli* translation system in 2013 [29]. In addition, a natural circRNA associated with rice yellow mottle virus was discovered to be capable of coding and translating proteins with overlapping initiation and termination codons (UGAUGA) [30]. We have demonstrated that the circRNAs produced from back-splicing of pre-mRNAs can be directly translated into functional proteins in human cells [13] and that circRNA translation can be driven by m6A modification [12]. Further investigations have identified several naturally translatable circRNAs with IRES elements, such as circZNF609 [ 31, 32], circMbl [33], circFGFR1 [34], circ-FBXW7 [35], and circFNDC3B [36]. These findings strongly support the translation of circRNAs in a cap-independent manner. Although the translation efficiency of circRNAs may be low under normal conditions, it can be increased by stress conditions such as heat/cold shock [12]. One possible reason may be that cap-dependent translation is inhibited under stress conditions, leading to more free ribosomes and translation-activating factors that can increase the translation efficiency of circRNAs [ 37, 38].

In addition to the IRES-driven mechanism, N6-methyladenosine (m6A), the most abundant internal base modification of RNA, can promote the efficient initiation of translation from circRNAs in human cells [12] ( Figure 1B). With the assistance of the m6A reader YTHDF3 and the initiation factor eIF4G2, a single m6A site is sufficient to drive translation initiation. Like the IRES-driven mechanism, the translation initiated by m6A shows low efficiency under normal conditions and is upregulated upon heat shock [12]. In support of this finding, circARHGAP35, which originates from the backsplicing of Exon 2 and Exon 3 of the tumor suppressor gene *ARHGAP35*, has also been observed as a translatable circRNA with m6A modification near the start codon. Notably, this circRNA is capable of being translated into proteins, contributing to the promotion of cancer cell progression [39] .

In addition to the IRES and m6A that can initiate circRNA translation, many IRES-like short elements (hexamers) have also been found to drive cap-independent circRNA translation via a cell-based screening system [14]. These IRES-like short elements are significantly enriched in endogenous circRNAs and can effectively drive circRNA translation ( Figure 1C). The majority of the identified translatable circRNAs code for new isoforms overlapping with canonical gene products, 50% of which can be translated into protein concatemers in a rolling circle fashion owing to the absence of a stop codon. Multiple *trans*-acting factors that bind these IRES-like elements to initiate translation have been identified [14].

All the translation mechanisms mentioned above highlight the versatility and complexity of circRNAs in generating novel protein products, which sheds light on the diverse functions and regulatory roles of circRNAs in various physiological changes and disease progression. In addition, artificially engineered circRNAs can also be designed to have different functions as protein-coding templates or noncoding regulatory RNAs, which will have a wide range of potential therapeutic applications as new drug modalities. The application of circRNA drugs involves the large-scale synthesis/purification of circRNAs and the efficient delivery of circRNAs to specific tissues, which will be further discussed in this review.

## *In Vitro* Synthesis of circRNAs

The first step of circRNA *in vitro* synthesis, similar to that of linear RNA, involves the utilization of RNA polymerase to transcribe RNAs from a linear DNA template [40]. The DNA templates usually contain the circRNA sequence and regulatory elements for RNA circularization. Additionally, efficient translation of *in vitro* transcribed circRNAs requires IRES elements, and the translation efficiency of circRNAs could be increased by optimizing the sequences of the ORF and IRES [ 34, 41]. Following *in vitro* transcription, linear precursor RNAs require further circularization and subsequent purification processes. *In vitro* transcription typically utilizes RNA polymerases derived from bacteriophages, such as T7 and SP6 RNA polymerases. The T7 RNA polymerase, which originates from the T7 bacteriophage, exhibits high specificity and efficiency in RNA synthesis, specifically recognizing the T7 promoter sequence for transcription initiation [42]. The optimized sequence TAATACGACTCACTATAGGGAGA is most commonly used for this purpose [42] .

For the synthesis of short circRNA sequences (< 100 nt), chemical ligation can be performed via cyanogen bromide (BrCN), which is an efficient chemical method that requires closely located reactive ends [43]. For longer sequences, circularization methods typically involve the use of RNA ligases and ribonucleases. Commonly used ligases include T4 DNA ligase (T4 Dnl), T4 RNA ligase 1 (T4 Rnl 1), and T4 RNA ligase 2 (T4 Rnl 2), all of which are ATP-dependent enzymes that catalyze the connection of RNA 5′-phosphate ends and 3′-OH ends through three nucleotide transfer steps to generate circRNAs [ 44 , 45].

**Figure 2:**
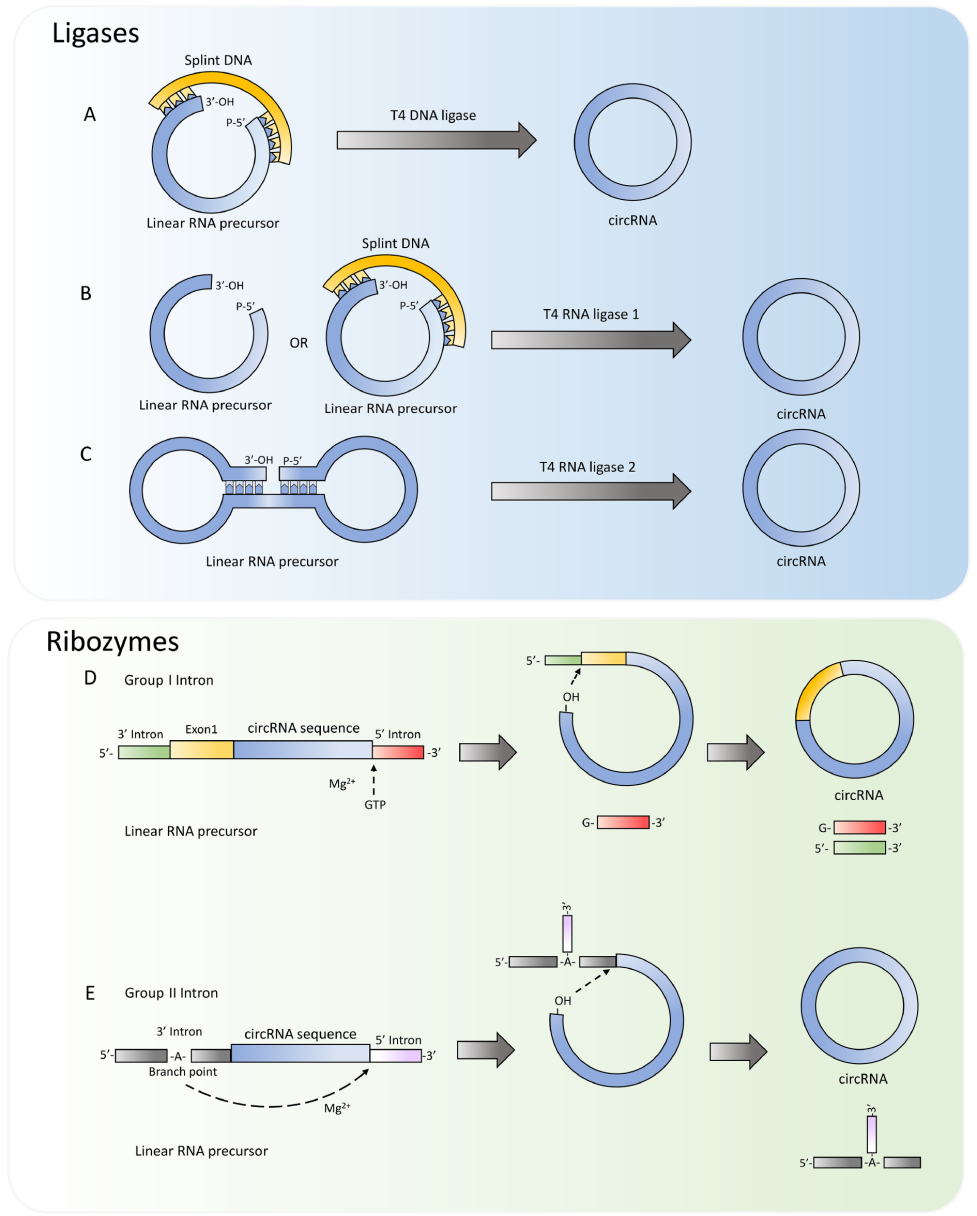
Figure 2 Circularization strategies for linear RNA precursors (A) Circularization strategy using T4 DNA ligases. (B) Circularization strategy using T4 RNA ligase 1. (C) Circularization strategy using T4 RNA ligase 2. (D) Circularization strategy using Group I introns. (E) Circularization strategy using Group II introns.

T4 DNA ligase can be used to connect RNA in addition to joining together DNAs, but it requires a double-stranded region for the ligation reaction. Therefore, a short nucleotide “splint” complementary to the target RNA is typically used to create the necessary double-stranded region, thereby catalyzing the connection of the 5′-phosphate end and the 3′-hydroxyl end of the precursor RNA ( Figure 2A). When T4 DNA ligase is used, the reaction conditions, including the temperature, amount of reactants, and splint length, need to be optimized to improve the circularization efficiency [45].

Unlike T4 DNA ligase, T4 RNA ligase 1 can catalyze the formation of a 3′-5′ phosphodiester bond in single-stranded RNA molecules and thereby can be used to circularize linear RNA [46] ( Figure 2B). Given that the ligation reaction mediated by T4 RNA ligase 1 is nonspecific, there is competition between intermolecular and intramolecular ligation. Therefore, it is necessary to optimize the reaction conditions to achieve circularization of the main reaction, such as by adding splint DNA to bring the two ends of an RNA molecule closer together. T4 RNA ligase 1 has different preferences for donor and acceptor nucleotides at the ligation site: for the 3′-end nucleotide acceptor, A > G ≥ C > U, and for the 5′-end phosphorylated nucleotide donor, pC > pU > pA > pG [ 47, 48].

T4 RNA ligase 2, which was tested in 2002, is also capable of catalyzing RNA circularization [49] ( Figure 2C). Compared with T4 RNA ligase 1, RNA ligase 2 can more efficiently ligate gaps on dsRNA substrates rather than on ssRNAs [50]. Using this feature, RNA ligase 2 is often used to circularize specific RNA structures where the donor and acceptor ends are brought close together by internal secondary structures [51].

When chemical methods or ligases are used for RNA ligation, the 5′-end of the RNA must be phosphorylated for the ligation reaction. Therefore, the triphosphate group at the 5′-end of RNA transcribed *in vitro* needs to be removed, followed by phosphorylation via a nucleotide kinase [52]. To simplify this step, GMP can be used instead of GTP during *in vitro* transcription with T7 RNA polymerase to obtain RNA with a 5′-monophosphate end for direct ligation [53]. However, this ligation process often requires splint sequences to close free ends to improve ligation efficiency. These methods present technical challenges in large-scale production because additional splint sequences and enzymes are used. Additionally, the use of ligases increases the complexity of product purification; therefore, this strategy is used mainly in research laboratories.

Alternatively, the ribonuclease method utilizes ribozyme sequences with self-splicing capabilities designed on both sides of the DNA template transcribed *in vitro*, resulting in the back-splicing of linear pre-circRNAs. Self-backsplicing mediated by Group I introns derived from Anabaena, also known as the permuted introns and exons (PIE) system, was first reported in 1992 and typically requires auxiliary exonic sequences, introducing additional sequences in circRNAs [54] ( Figure 2D). The addition of GTP and Mg ^2+^ are required in this method as cofactors to catalyze the circularization process. During the reaction, the free GTP added attacks the splice site at the 5′ end of the downstream intron and breaks it. The 3′-hydroxyl group generated at the end of the exon (E1) then attacks the splice site at the 3′-end of the upstream intron, directly linking the two exons in a 3′-5′ phosphodiester bond, forming a circular RNA while simultaneously removing the excess intron. The circular products obtained by this method have high uniformity, but a fraction of the end sequences in the exons are retained in the final product (usually from bacterial or bacteriophage sources), which may distort the desired conformation of circular RNAs and lead to significant immunogenicity [55].

In 2018, the group of Daniel G. Anderson achieved the circularization of long coding RNA *in vitro* by engineering and modifying the Group-I intron of Anabaena [18]. They increased the number of homology arms on the intron to bring the splice sites closer spatially, thereby improving the circularization efficiency. Considering that the splice site of the 3′ intron is close to the IRES element and that both sequences are highly conserved and structured, a spacer sequence was designed to prevent interference in space. As a result, long RNAs can be circularized with greater efficiency via this method, although circular RNAs contain some exogenous sequences. This study also demonstrated for the first time that engineered circular RNAs produced *in vitro* can stably and efficiently express proteins in eukaryotic cells, validating circular RNAs as effective alternatives to linear mRNAs. A recent study in 2024 reported that shortening the sequence of homology arms generates a new version of PIE by a 27-nt extraneous fragment in final circular RNA products with high yield and low immunogenicity [56] .

In 2022, a novel circularization strategy based on Group I introns called Clean-PIE was reported [57]. In this study, the optimal circularization sites were optimized by screening the coding region of the target protein or IRES region, achieving precise and efficient circularization of RNA without introducing exogenous sequences (circularization rate > 90%). Additionally, this study established an efficient screening system for IRES elements, obtaining multiple IRES sequences with expression performance superior to that of CVB3. The circRNAs obtained through the Clean-PIE strategy have the advantages of high expression efficiency, low immunogenicity, and a long duration of expression, laying the foundation for further large-scale production and clinical applications.

Comparatively, self-backsplicing facilitated by Group II introns from yeast mitochondrial genomes does not require auxiliary exon sequences, yielding the anticipated circRNA sequence [ 58, 59] ( Figure 2E). In this case, we designed an artificial ribozyme that can better simulate the RNA splicing process in eukaryotes. This circularization process does not require additional GTP and can significantly shorten the lengths of E1 and E2 by retaining only the intron binding site (IBS) recognized by the exon binding site (EBS). Furthermore, a portion of the coding region sequence can be used as the IBS sequence, and appropriate mutations and modifications can be made to the EBS sequence of the intron to reduce the immunogenicity caused by introducing exogenous sequences while ensuring high circularization efficiency. Additionally, this strategy involves mild reaction conditions and can be carried out in the form of cocircularization during *in vitro* transcription, greatly simplifying the production process [60].

During the production of circular RNA, there may also be additional byproducts, such as unspliced linear RNA precursors and excised introns, which can be separated and purified via high-performance liquid chromatography (HPLC) [19].

## *In Vivo* Synthesis Strategy for circRNA Drugs

By introducing a DNA plasmid template into cells, the *in vivo* strategy utilizes the cells as biological reactors to synthesize the target RNA precursors, which subsequently undergo splicing within the cells to produce circRNAs. However, this strategy is limited by subsequent purification issues, as various RNA molecules inside the cells make it difficult to purify the synthesized circRNA. Therefore, *in vivo* synthesis is often associated with the engineering of cell-based delivery vectors, where circRNAs generated within cells are subsequently encapsulated in the cells as viruses or exosomes and excreted from the cells. This strategy enables the direct collection of circRNAs encapsulated in delivery vectors. For example, the Tornado (Twister-optimized RNA for durable overexpression) expression system, which is based on tRNA autocatalytic splicing, was invented in 2019 to achieve rapid RNA circularization, resulting in highly efficient expression of circular RNA aptamers in cells [61]. The plasmids in the tornado expression system are transcribed to RNAs flanked by Twister ribozymes, which rapidly undergo autocatalytic cleavage, leaving termini that are ligated by the ubiquitous endogenous RNA ligase RtcB.

## Delivery of circRNAs

**Figure 3:**
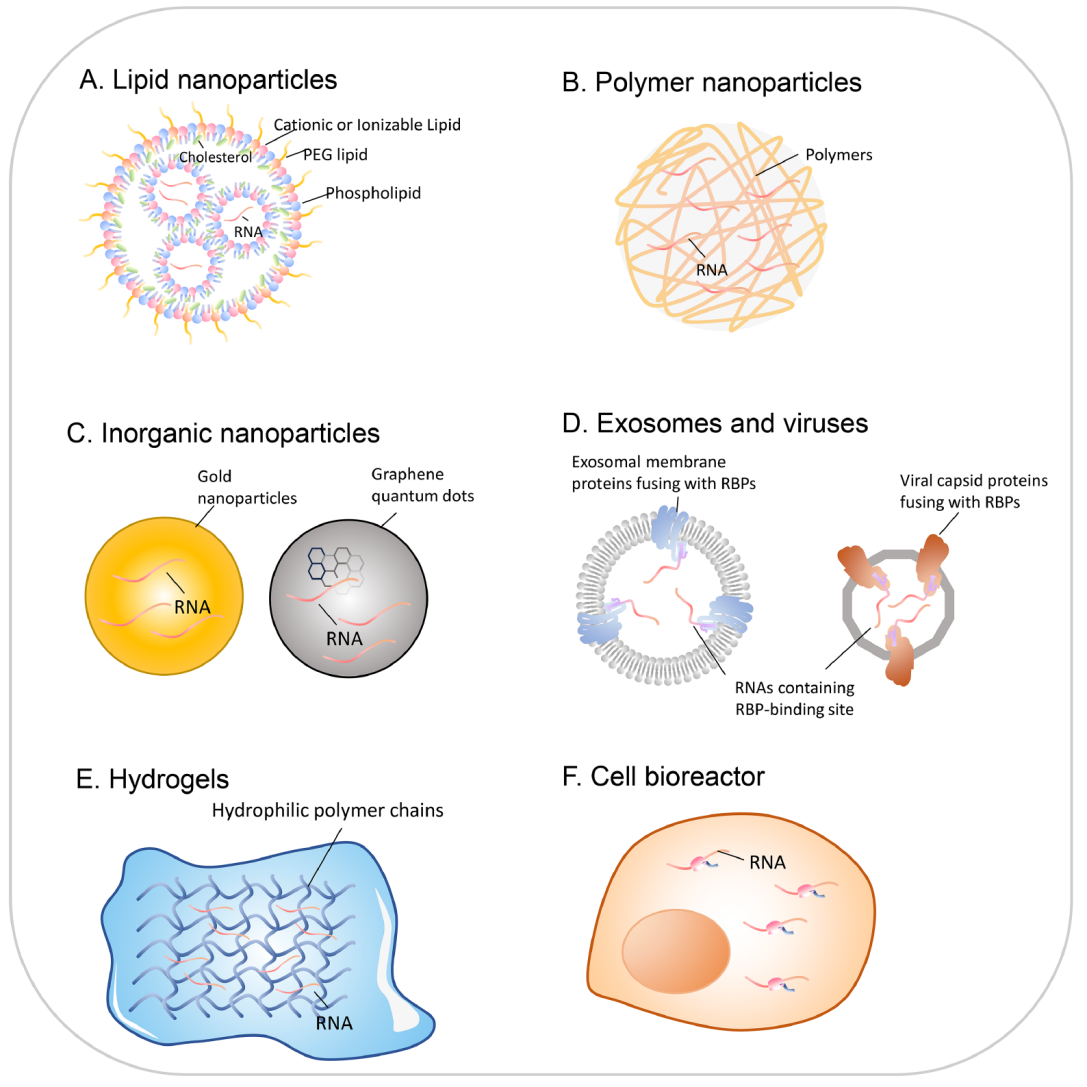
Figure 3 Various delivery tools that can be used for circRNA-based drugs

Because RNA is a large anionic molecule that has difficulty passing through cell membranes, let alone specific targeting, the effective delivery of circRNAs into target cells is another key focus and challenge for circRNA-based biomedical applications. Currently, extensive research on vectors for packaging and delivering RNA has been carried out, ranging from nanoscale to microscale, from *in vitro* synthesis to *in vivo* cell secretion ( Figure 3). A wide variety of delivery tools, such as nanolipid particles, polymer nanoparticles, exosomes, viruses, and hydrogels, have been tested for circRNA delivery. In principle, circRNAs can be delivered via all strategies used for other types of RNAs, which are summarized below.

Currently, the most widely studied and utilized tool for RNA delivery is lipid nanoparticles (LNPs). LNP formulations typically consist of four key components: ionizable lipids, phospholipids, cholesterol, and polyethylene glycol (PEG) lipids ( Figure 3A). Cationic lipids with a positive charge can form complexes with RNA and facilitate entry into various cells [62]. However, cationic lipids exhibit high cytotoxicity since they may interact with key enzymes within cells, such as protein kinase C, to affect their functions. Additionally, cationic lipids also have high immunogenicity. The intravenous injection of cationic liposomes promotes the release of type I and type II interferons, stimulating an immune response [63]. Therefore, ionizable lipids are used to construct lipid nanoparticles for RNA delivery, since the charge of the hydrophilic head of the ionizable lipids can change with pH. They are positively charged in acidic environments to promote the formation of cationic liposomes that bind with RNA but become neutral in physiological environments to reduce toxicity and immunogenicity and extend their presence *in vivo*. Finally, as LNPs are taken up by cells and subsequently enter the endosomal environment, they undergo charging reprogramming to promote the release of RNA [64]. Commonly used ionizable lipids include SM-102 and ALC-0315, among others [62]. Phospholipids can promote LNP stability and fusion with cell membranes and endosomal membranes, facilitating cellular uptake and intracellular RNA release. The commonly used phospholipids in LNP formulations are 1,2-distearoyl-sn-glycero-3-phosphorylcholine (DSPC) or 1,2-dioleoyl-sn-glycero-3-phosphoethanolamine (DOPE). Cholesterol effectively enhances LNP stability and promotes fusion with cell membranes. PEG forms a hydrophilic barrier around LNPs, suppressing aggregation and reducing immunogenicity by preventing protein-LNP binding in the blood, thereby prolonging the presence of LNPs *in vivo* [65].

Nanoparticles made of cationic polymers exhibit characteristics similar to those of cationic lipid nanoparticles ( Figure 3B) but also show high cytotoxicity. Recent research has focused more on biocompatible polymer nanoparticles such as PBAEs [66]. Another type of charge-changeable polymer nanoparticle (CART) can release mRNA into cells by rearranging the charges within the particles, indicating promising applications [67].

The LPP (lipopolyplex) nano-delivery platform is a bilayer structure with polymer-loaded RNA as the core and lipid wrapping as the shell. It encapsulates the target RNA within a double-layered shell of lipids to protect the RNA molecules from degradation by RNase [68]. LPP combines the advantages of polymers and liposomes, demonstrating good stability, low cytotoxicity, high gene transfection efficiency, and slow release of the mRNAs packaged during polymer degradation. For example, the excellent dendritic cell targeting ability of the LPP platform was used to activate the T-cell immune response through antigen presentation, aiming to achieve the desired immunotherapeutic effect in cancer vaccines [ 68, 69].

Compared with polymer nanoparticles, inorganic nanoparticles have advantages such as ease of synthesis, controllable size, high biocompatibility, and well-established properties for research ( Figure 3C). Currently, gold nanoparticles are widely used for DNA and siRNA delivery because of their excellent safety and structural stability [ 70, 71]. To date, polymer-modified gold nanoparticles, polyethyleneimine-modified graphene quantum dots, and selenium nanoparticles have been used to deliver mRNA [ 72– 74]. Although these inorganic nanoparticles have excellent mRNA delivery capabilities, their loading efficiency is relatively low because mRNA is loaded on the surface of the nanoparticles. To overcome this problem, multifunctional silica nanoparticles, which are reported to have high mRNA packaging rates, smaller sizes, diverse surface modification capabilities, and efficient endosomal escape and release characteristics, have been used for mRNA delivery [75].

However, the long-term nonspecific accumulation of inorganic nanomaterials in the human body may lead to toxicity, hindering their large-scale clinical application. Therefore, clinical toxicological evaluations of this delivery strategy are critical. In addition, developing new biodegradable or clearable inorganic nanomaterials is another direction for addressing this issue. Recently, organic‒inorganic hybrid nanoparticles (mesoporous silica nanoparticles coated with cationic polymers) have been designed through iterative screening for *in vivo* mRNA delivery and are biodegradable [76]. The target mRNA is premixed with cationic polymers such as polyethyleneimine (PEI) and then electrostatically bound to the surface of mesoporous silica nanoparticles (MSNPs). The resulting particles exhibited excellent mRNA transfection efficiency both *in vitro* and *in vivo* .

In addition to various strategies for the *in vitro* synthesis of delivery vectors, the use of cells to produce viruses and exosomes is also a commonly studied delivery strategy ( Figure 3D). Delivery vectors based on viruses typically involve transfecting cells with two plasmids—one encoding viral capsid proteins that assemble in cells and fuse with a specific RNA-binding protein and another plasmid for transcribing the target RNA containing the binding site for this RNA-binding protein [77]. This enables the target RNA to be loaded into the virus and secreted externally. In a recent study, the research group led by Samie R Jaffrey utilized this delivery strategy to achieve cell-specific delivery of circRNAs produced by the Tornado expression system. However, the system also faces the issue of nonspecific encapsulation [78] .

In 2021, the group led by Feng Zhang [79] developed a new method called SEND (selective endogenous eNcapsidation for cellular delivery) for delivering biomolecules to cells via human proteins. This system utilizes naturally occurring protein particles that resemble viruses and can bind to RNA binding proteins (PEG10) for delivery, theoretically resulting in lower immunogenicity. This system can be programmed to package and transport different types of RNA, which may provide an endogenous delivery carrier for RNA-based gene therapy.

Exosomes are extracellular vesicles of approximately 100 nm in size that are secreted by cells. Similar to virus-based vectors, delivery of RNAs by exosomes involves transfecting cells with two plasmids—one expressing exosomal membrane proteins such as CD63, which is fused with a specific RNA-binding protein (RBP), and another plasmid for the transcription of the target RNA containing the corresponding RBP-binding sequence. The RNA is then enriched in exosomes and secreted externally [80]. Furthermore, surface functionalization of exosomes can control cellular immunity and target specific cells or tissues [ 81, 82]. Modified exosomes have the potential to precisely deliver mRNA molecules to target cells or organs. While these methods harness the targeting and biocompatibility advantages of viruses and exosomes, *in vivo* synthesis may result in lower RNA purity than *in vitro* strategies and could be challenging to purify.

In addition to nanoscale delivery vectors, hydrogels, networks consisting of hydrophilic polymer chains that exhibit water absorption characteristics and similar properties to those of the extracellular matrix, can also serve as RNA delivery vectors [83]. Hydrogels can load naked RNA or RNA encapsulated in nanoparticles, thus enabling the controlled release of RNA to local tissues ( Figure 3E). The sustained release of cargo molecules by hydrogels may last for several weeks. RNA diffuses into neighboring tissue cells passively or via degradation and swelling of the hydrogel, and the release rate of RNA is controlled by adjusting parameters such as the molecular weight of the hydrogel, matrix concentration, cross-linking density, pore size distribution, and coupling between RNA and hydrogel molecules. Furthermore, by adjusting the composition of the hydrogel, smart-responsive RNA release hydrogels can be constructed in response to factors such as pH, enzymatic cues, and light. There is great potential to apply hydrogels as new RNA delivery vectors in tumor therapy, bone regeneration, cardiac repair, and wound healing, among other applications.

In addition to common delivery strategies, some researchers combine cell therapy with RNA therapy. In this case, cells transfected with target RNAs as biological reactors may be injected into patients (or animal models) to achieve the effect of protein replacement therapy ( Figure 3F). For example, skin fibroblasts pretreated with *in vitro* transcribed mRNA expressing vascular endothelial growth factor (VEGF) were administered to mice, which significantly reduced tissue necrosis and dramatically improved vascular density in a murine model of critical limb ischemia (CLI) [84].

## Biomedical Applications of circRNAs

### Applications as translatable RNAs

Given that *in vitro* transcribed circRNAs can be translated in a cap-independent fashion and show greater stability and lower immunogenicity, circRNAs can be seen as linear mRNAs with special structures, which has various biomedical applications, including vaccines and protein replacement therapy. Here, we briefly summarize the biomedical applications of translatable circRNAs as a special form of mRNA. For more detailed information on recent research advancements in mRNA therapy, please refer to other comprehensive reviews [85].

One of the most popular applications for circRNAs and mRNAs is to express antigens as vaccines after the emergence of COVID-19 mRNA vaccines. A circRNA vaccine encoding the trimeric RBD antigen of the SARS-CoV-2 spike protein was reported in 2022 [86]. Owing to the high stability of circRNAs, this vaccine induces more and more durable antigens to elicit a greater proportion of neutralizing antibodies and distinct Th1-skewed immune responses than the 1mΨ-modified mRNA vaccine does and effectively protects against SARS-CoV-2 in mice and monkeys [86].

Additionally, circRNAs can also be used as cancer vaccines in cancer immune therapy. The application of circRNAs as cancer vaccines was first reported in 2022, in which circRNAs exhibited more durable protein expression ability than modified linear mRNAs did, triggered robust innate and adaptive immune responses, and showed antitumor efficacy in multiple mouse tumor models [87]. Recently, HLA-I-binding cryptic antigenic peptides that are noncanonically translated by a tumor-specific circRNA, circFAM53B, were identified via mass spectrometry of the HLA class I (HLA-I) peptidome coupled with ribosome sequencing of human breast cancer samples [88]. This finding reveals that peptides translated by tumor-specific circRNAs have the potential to drive efficient antitumor immunity if they have HLA-I-binding abilities. The administration of vaccines consisting of tumor-specific circRNAs or their encoded peptides in mice bearing breast cancer or melanoma induced enhanced infiltration of tumor-antigen-specific cytotoxic T cells, which effectively controlled tumor growth.

In addition to being used in vaccines, translatable circRNAs can also be used in protein replacement therapy, which usually requires a much higher level of protein production. mRNA-based protein replacement therapy uses the biological properties of mRNAs to express proteins that are defective or missing in certain diseases. Compared with protein-based therapies, mRNA therapy enables the expression of functional proteins in the cytoplasm and the ability to target cellular membranes. Additionally, compared with DNA-based therapies, mRNA therapy avoids the nuclear entry step, making it easier to deliver and decreasing the risk of integrating foreign DNAs into the genome. Currently, there are extensive studies on mRNA therapy for protein replacement. For example, it has been used in targeting metabolic disorders such as phenylketonuria [89], Fabry disease [90], and glycogen storage disease type Ia [91] caused by defects in related metabolic enzyme-encoding genes. Furthermore, mRNA therapy can also regulate cell growth and differentiation through the expression of cytokines. For example, mRNA encoding VEGF-A was used to promote myocardial cell renewal for treating myocardial infarction in mouse models [92].

To date, there is still no protein replacement therapy involving circRNAs. However, we can appreciate its potential through studies on mRNAs, considering the greater stability and lower immunogenicity of circRNAs than linear mRNAs. Exogenous circRNAs may evade RNA sensors, including RIG-I and TLRs, and exhibit more durable protein expression than linear mRNAs in mice [19]. These results contrast with those of earlier work in 2017, which showed that circRNAs provoke a strong innate immune response mediated by RIG-I [93], which may be caused by the impurity of circRNAs, since the RNase R used in previous work may not be sufficient to obtain pure circRNAs.

Recently, a programmable miRNA-responsive IRES translation activation and repression (PROMITAR) platform was developed [94]. This platform enables specific miRNAs to combine with the pseudoknot of the IRES, and this modulation of the IRES structure will upregulate or downregulate circRNA translation. Moreover, by relying on different translation initiation factors required by different IRES sequences, the use of different IRES sequences allows circRNAs to have the potential for cell-specific translation in different cells.

### Applications of circRNAs as noncoding RNAs

Since circRNAs are also RNA molecules present in living organisms, with a deeper understanding of the mechanisms of endogenous circRNA molecules in recent years, research on the intrinsic functions of circRNAs for therapeutic purposes has become increasingly widespread. Because circRNAs function as miRNA sponges, an artificial circRNA containing repeated binding sites for miR-21was synthesized in 2018 to treat gastric carcinoma, in which miR-21 is highly expressed. The synthesized circRNA functions as a miR-21 sponge; thus, it suppresses the function of miR-21 and suppresses GC proliferation [95]. An artificial circRNA containing 12 binding sites for 2 miRNAs was constructed to competitively bind to miR-132 and miR-212, which are expressed during cardiac hypertrophy. *In vivo* circRNA administration via the AAV9 vector attenuated disease in a mouse model [96]. Artificial circRNAs can also be designed as sponges for proteins. circRNAs with CA-repeat or CA-rich sequences are synthesized as protein sponges to specifically bind and functionally inactivate hnRNPL (heterogeneous nuclear ribonucleoprotein L) , which is a type of protein that regulates alternative splicing, depending on short CA-rich RNA elements [97].

IVT-generated circRNAs can also be used in RNA editing as guide RNAs. For example, circRNAs, termed circ-arRNAs, are used as engineered ADAR-recruiting RNAs to recruit endogenous ADAR enzymes in programmed RNA editing, which results in much higher editing efficiency than their linear counterparts when expressed in cells or delivered to mice [98] .

In addition, Liu *et al*. [55] reported that extra fragments in circRNAs introduced by Group I introns provoked immune responses, whereas those produced by T4 RNA ligase exhibited minimal immunogenicity and were able to form short dsRNA regions to efficiently suppress PKR activation. An artificial circRNA was designed to act as an antisense RNA targeted to SARS-CoV-2 RNA, which resulted in up to 90% reduction in virus proliferation in cells and lasted at least 48 h [99]. Finally, as RNA aptamers, a circRNA-based fluorescent metabolite biosensor for S-adenosyl methionine (SAM) is much more efficient and more durable than linear RNAs are, which makes circRNA-based biosensors sufficient for the detection of intracellular SAM dynamics when expressed at low levels [61].

## Challenges for circRNA As a New Drug Modality

Although their stability and low immunogenicity provide certain advantages for the biomedical application of circRNAs, the application of circRNAs still faces many challenges.

First, a major limitation of current RNA therapy is the lack of highly effective delivery vectors, especially for protein replacement therapies that require high protein expression levels. On the one hand, the various delivery methods currently available have significant differences in their ability to target different organs, which makes it more difficult to target certain organs, such as the brain, kidneys, and spleen, and, in turn, increases the difficulty of developing therapies for diseases related to these organs. Even in organs that are relatively easy to target, such as the skin, eyes and liver, another challenge is how to selectively target specific cell types in these organs. On the other hand, delivery vectors such as LNPs still have immunogenicity issues, which pose a major challenge for protein replacement therapies that may require long-term or lifelong administration. The immune response to the delivery vector reduces its half-life within the body and may also lead to various toxic and adverse reactions.

Additionally, it is necessary to construct suitable circRNA sequences that can express the target protein at high levels or perform the anticipated functions with greater efficiency. Research on the IRES sequence responsible for translation initiation still has many unknown aspects, and the numerous noncoding functions of circRNAs have yet to be discovered. Essentially, we still know very little about the relationships among the sequences, structures, and functions of RNA. Moreover, we currently lack powerful AI tools such as AlphaFold3 for protein design to predict or optimize RNA sequences.

Finally, there is still a challenge in using appropriate synthesis and production strategies to purify these materials in large quantities. Both ligase-catalyzed and ribozyme-catalyzed circularization reactions produce additional byproducts that may lead to additional adverse effects. The purification of various byproducts of circRNA production during preparation still cannot be achieved perfectly through HPLC alone. In addition, balancing the output, purity, and cost of circRNA production is a significant challenge for successful clinical translation.

## Summary and Outlook

Different circRNAs have their own unique functions *in vivo*, and together with other biological macromolecules, such as proteins, they form a complex network that regulates life activities. By synthesizing circRNAs *in vitro*, we can utilize the diverse functions of circRNAs for applications in various fields of biomedicine. Owing to their improved stability and lower immunogenicity, circRNAs are more advantageous for exerting long-lasting effects *in vivo* and reducing toxic side effects. Additionally, for translatable circRNAs, further screening and optimization of the IRES sequence responsible for translation initiation are expected to increase the translation efficiency of circRNAs and may provide the ability of circRNAs to exhibit cell-specific expression in different cell types. Another key problem in RNA therapy is delivery: by selecting appropriate delivery vectors and administration methods for different therapeutic organs, advancements in delivery vector research will inevitably promote the development of circRNA therapy. Overall, increased delivery efficiency, increased expression efficiency, and decreased immunogenicity are key directions for the future development of circRNA technology.

Researchers successfully expressed target proteins by injecting naked mRNAs into mouse muscle tissue as early as 1990. Over the next 30 years, with the continuous discovery of the physiological functions and molecular mechanisms of RNAs, breakthroughs in *in vitro* synthesis processes and drug delivery technologies have allowed the use of mRNA vaccines to combat the COVID-19 pandemic. Similarly, various applications based on circRNAs are beginning to be considered for clinical use only recently; therefore, the accumulation and optimization of various technologies from basic research to clinical applications are needed. While the concept of circRNA therapy is straightforward, it requires the accumulation and optimization of various technologies from basic research to clinical applications, and the absence of any key technology may result in poor treatment outcomes. Continuous and in-depth research is still needed on the biogenesis, biological functions, and degradation of circRNAs. Without discoveries on the physiological mechanisms of circRNAs, such as ceRNA activity and translation, the current biomedical applications of circRNAs are not possible. More data about the relationships among RNA sequences, structures and functions are also beneficial for the development of effective AI prediction tools to accelerate the development of this field. After 4 billion years of evolution, nature has provided us with an endless treasure trove waiting to be explored. New discoveries will undoubtedly further promote the biomedical applications of circRNAs.
